# Characterization of α-Glucosidase Inhibitors from *Clinacanthus nutans* Lindau Leaves by Gas Chromatography-Mass Spectrometry-Based Metabolomics and Molecular Docking Simulation

**DOI:** 10.3390/molecules23092402

**Published:** 2018-09-19

**Authors:** Suganya Murugesu, Zalikha Ibrahim, Qamar-Uddin Ahmed, Nik-Idris Nik Yusoff, Bisha-Fathamah Uzir, Vikneswari Perumal, Faridah Abas, Khozirah Saari, Hesham El-Seedi, Alfi Khatib

**Affiliations:** 1 Department of Pharmaceutical Chemistry, Kulliyyah of Pharmacy, International Islamic University Malaysia, Kuantan 25200, Pahang Darul Makmur, Malaysia; suganya.murugesu@gmail.com (S.M.); zalikha@iium.edu.my (Z.I.); qamaruahmed@yahoo.com (Q.-U.A.); nikzed@yahoo.com (N.-I.N.Y.); bishafu@iium.edu.my (B.-F.U.); 2 Faculty Pharmacy & Health Sciences, Universiti Kuala Lumpur Royal College of Medicine Perak, Ipoh 30450, Perak Darul Ridzuan, Malaysia; vikneswari@unikl.edu.my; 3 Laboratory of Natural Products, Institute of Bioscience, Universiti Putra Malaysia, Serdang 43300, Selangor Darul Ehsan, Malaysia; faridah_abas@upm.edu.my (F.A.); khozirah@upm.edu.my (K.S.); 4 Division of Pharmacognosy, Department of Medicinal Chemistry, Biomedical Centre, Uppsala University, Box 574, SE-751 23 Uppsala, Sweden; hesham@kth.se; 5 H.E.J. Research Institute of Chemistry, International Centre for Chemical and Biological Sciences, University of Karachi, Karachi 75270, Pakistan

**Keywords:** α-glucosidase inhibitors, *Clinacanthus nutans*, diabetes, GC-MS, partial least square

## Abstract

Background: *Clinacanthus nutans* (*C. nutans*) is an *Acanthaceae* herbal shrub traditionally consumed to treat various diseases including diabetes in Malaysia. This study was designed to evaluate the α-glucosidase inhibitory activity of *C. nutans* leaves extracts, and to identify the metabolites responsible for the bioactivity. Methods: Crude extract obtained from the dried leaves using 80% methanolic solution was further partitioned using different polarity solvents. The resultant extracts were investigated for their α-glucosidase inhibitory potential followed by metabolites profiling using the gas chromatography tandem with mass spectrometry (GC-MS). Results: Multivariate data analysis was developed by correlating the bioactivity, and GC-MS data generated a suitable partial least square (PLS) model resulting in 11 bioactive compounds, namely, palmitic acid, phytol, hexadecanoic acid (methyl ester), 1-monopalmitin, stigmast-5-ene, pentadecanoic acid, heptadecanoic acid, 1-linolenoylglycerol, glycerol monostearate, alpha-tocospiro B, and stigmasterol. In-silico study via molecular docking was carried out using the crystal structure *Saccharomyces cerevisiae* isomaltase (PDB code: 3A4A). Interactions between the inhibitors and the protein were predicted involving residues, namely LYS156, THR310, PRO312, LEU313, GLU411, and ASN415 with hydrogen bond, while PHE314 and ARG315 with hydrophobic bonding. Conclusion: The study provides informative data on the potential α-glucosidase inhibitors identified in *C. nutans* leaves, indicating the plant’s therapeutic effect to manage hyperglycemia.

## 1. Introduction

Diabetes mellitus (DM) is one of the most chronic metabolic disorders that occurs due to abnormal carbohydrate metabolism that is mainly linked with insensitivity of the target organs to insulin [1,2]. Commonly, it is characterized by hyperglycemia in which the level of blood sugar is significantly elevated, which in turn disturbs the normal body metabolism related to carbohydrate, fat, and protein breakdown and eventually proves fatal if not treated or managed properly [3]. Cases of DM are increasing rapidly worldwide and affecting all parts of the world. High increasing numbers of cases have been reported in most developing countries, including Malaysia; caused mainly by inactive lifestyle and unhealthy food consumption [4]. Around 170 million people worldwide had diabetes in the year of 2000 and it is projected to increase to 366 million by 2030 [5]. Additionally, the prevalence of DM is also rapidly rising among the middle-class and low-income communities in developing countries [6].

The use of several traditional medicinal plants in the treatment of diabetes has been reported [7,8]. Likewise, *Clinacanthus nutans* (Burm. F) Lindau; (*C. nutans*) or Sabah snake grass (locally known as ‘belalai gajah’), is a herbal shrub found abundantly in some Asian countries including Thailand and Malaysia. This herb has been traditionally used to treat various diseases including gout, hyperuricemia, inflammation, fever, skin rashes, and diabetes [9]. Commonly, the plant leaves are used for the treatment of burns, allergic reactions, mucositis, skin rashes, and lesions of varicella-zoster virus (VZV) and herpes simplex virus (HSV) [10,11]. In Malaysia and Thailand, it has been practiced as a remedy for scorpion and insect stings as well as venomous snake bites [12,13]. In recent times, the leaves of *C. nutans* are widely consumed by cancer patients throughout Malaysia [14] while in Indonesia, the leaves are used to treat diabetes, hyperuricemia, and dysentery [15,16].

*C. nutans* leaves are reported to contain several compounds that possess hypoglycemic properties such as caffeic acid, chlorogenic acid, quercetin, lupeol, betulin, stigmasterol, and β-sitosterol [17,18,19,20]. Most of the existing studies were carried out separately to evaluate the biological activities of some individual compounds. However, the bioactivity of the individual compounds may be different when these compounds are within the complex matrix of the leaves, which could be due to the synergistic effect with other components [21]. Thus, the reported antidiabetic compounds may not be solely responsible for the actual antidiabetic activity of the plant. Therefore, one way of detecting the compounds responsible for a given activity is by using the metabolomics approach. It is a holistic approach that includes the detection of all metabolites in each sample that can be correlated to the tested bioactivity using multivariate data analysis (MVDA) [22]. Metabolomics techniques can be achieved with the aid of chromatography or spectroscopy instruments to profile all the metabolites present in the analyzed sample. MVDA is a suitable statistical tool for managing large data sets obtained using spectroscopic tools and is employed in classifying samples based on their chemical constituents [23]. 

Several common analytical instruments have been used as an analytical platform in metabolomics, including nuclear magnetic resonance spectroscopy (NMR), gas chromatography-mass spectrometry (GC-MS), and liquid chromatography-mass spectrometry (LC-MS) [24]. In this study, GC-MS was utilized due to the non-polar nature of the most active hexane extract. Moreover, GC-MS provides high resolution of separation, high sensitivity, and the reproducibility needed to analyze a complex biological mixture [25]. Hence, the aims of this study were to evaluate the α-glucosidase inhibitory activity of different extracts of *C. nutans* leaves, and to identify the related bioactive compounds in the extract using GC-MS based metabolomics. 

The study has also depicted the molecular interaction between the enzymes and the inhibitors (ligands) identified in the plant extracts. With molecular docking, the conformations and binding affinities of the potential phytoconstituents could be predicted. In addition, this in-silico technique aids in the visualization of the ligand-protein complex formed while portraying the characteristic activity and binding sites of the compounds [26].

## 2. Results 

### 2.1. α-Glucosidase Inhibitory Activity

The α-glucosidase inhibitory activity of the *C. nutans* leaves extracts obtained from different solvent extraction are displayed in Table 1 as the half-maximal concentration values (IC_50_, μg/mL). A lower IC_50_ value is desirable for higher α-glucosidase inhibition activity [27]. The IC_50_ values ranged from 3.07 to 133.57 μg/mL. The highest α-glucosidase inhibitory activity was observed for hexane fraction, with an IC_50_ value of 3.05 μg/mL, which was found comparable or even better to the IC_50_ value obtained for quercetin, the positive control. Meanwhile, the methanol extract exhibited the lowest activity with the highest IC_50_ viz., 133.57 μg/mL. 

### 2.2. GC-MS Chromatogram of C. nutans Leaves Extracts

**Figure 1:**
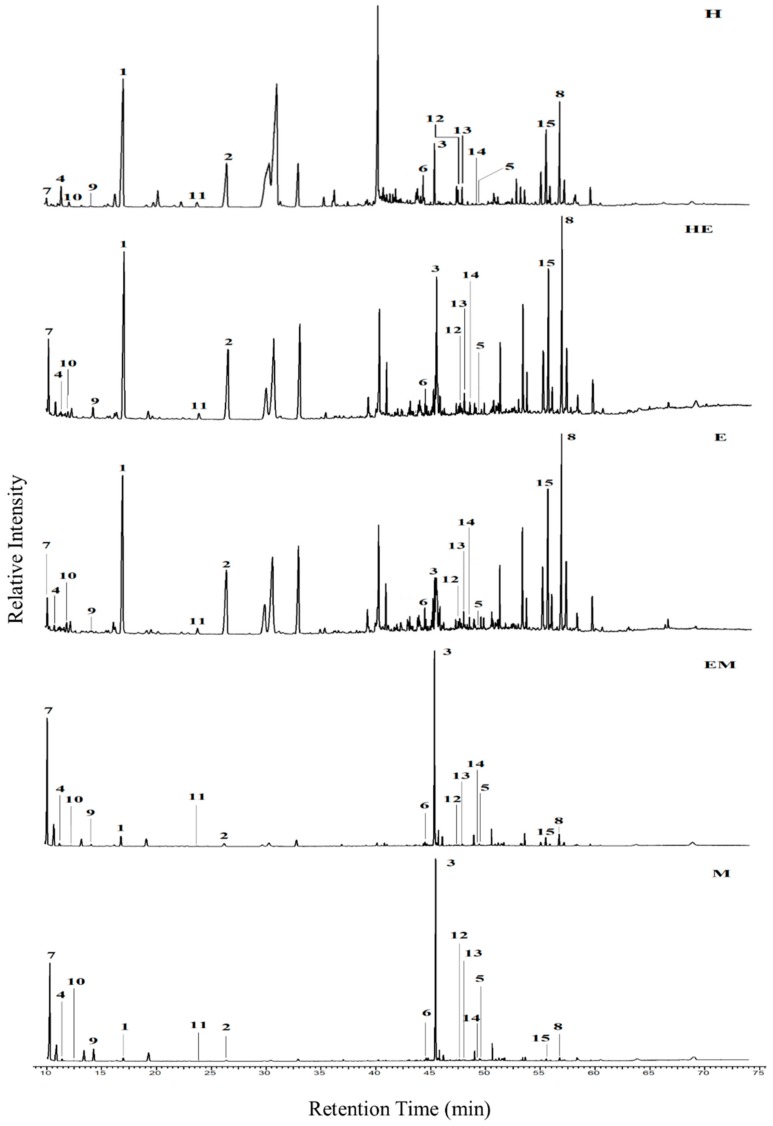
GC-MS chromatogram of metabolites in H, HE, E, EA and M extracts from *C. nutans*. Each labelled peak refers to; **1**—palmitic acid, **2**—phytol, **3**—sucrose, **4**—hexadecanoic acid, **5**—maltose, **6**—1-monopalmitin, **7**—d-glucose, **8**—stigmast-5-ene, **9**—d-gluconic acid, **10**—pentadecanoic acid, **11**—heptadecanoic acid, **12**—1-Linolenoylglycerol, **13**—Glycerol monostearate, **14**—Alpha-Tocospiro B and **15**—Stigmasterol.

All identified metabolites were confirmed by scrutinizing the spectral pattern and comparison with GC-MS NIST14 database library. The metabolites identified were primarily plant sterols, fatty acids, organic acids, and others, including palmitic acid, phytol, hexadecanoic acid, 1-monopalmitin, stigmast-5-ene, pentadecanoic acid, heptadecanoic acid, 1-linolenoylglycerol, glycerol monostearate, alpha-tocospiro B and stigmasterol. The remaining compounds include sucrose, maltose, d-gluconic acid and d-glucose, which were abundant in the non-active extract (methanol extract). All the above-mentioned metabolites are labelled and shown in the chromatogram of each of the extract analyzed, as displayed in Figure 1. Several compounds are found abundantly in H, HE and E extracts especially palmitic acid, phytol, stigmast-5-ene and stigmasterol. The intensity of the metabolites reduced apparently as the polarity of the extract solvent increased. Meanwhile, sucrose and d-glucose were found more prominent in the more polar extract (EM and M). 

### 2.3. Multivariate Data Analysis

The bioactive compounds can be identified through GC-MS based metabolomics utilizing MVDA whereby a partial least square (PLS) model was developed. Automated fitting results in two principal components namely PLS component 1 and 2. The mass to charge ratio (*m*/*z*) of a certain retention time was extracted and the IC_50_ (µg/mL) values of each of the samples were considered as x and y variables, respectively. PLS was performed to discriminate the constituents among the extracts and to pinpoint the *m*/*z* values contributing to the bioactivity [1]. Briefly, the model diagnostic of the PLS model developed can be evaluated using several parameters such as goodness of fit, permutation test, and capability of the model to predict the y value using actual to predicted plot. The goodness of fit is explained by the cumulative values of R^2^Y indicating the percentage of variation of the response explained by the model, and the cumulative value of Q^2^Y representing the percentage of the variation of the response predicted by the model according to cross validation. The fitness of model and predictive ability are considered good if the cumulative values of both R^2^Y and Q^2^Y are greater than 0.5 [28]. In this study, the R^2^Y and Q^2^Y values were 0.95 and 0.87, respectively. 

Other important parameters that are considered in measuring the accuracy and the performance of the model are root-mean-square errors of estimation (RMSEE) and the root mean square error of cross-validation (RMSECV). RMSEE is a measure of average deviation of the model from the data, while RMSECV is a measure of the quality of the model in predicting new samples. The lower the RMSECV value, the higher the predictive accuracy for new samples. For this model, the RMSEE and the RMSECV values are 0.0286046 and 0.0441324, respectively. 

**Figure 2:**
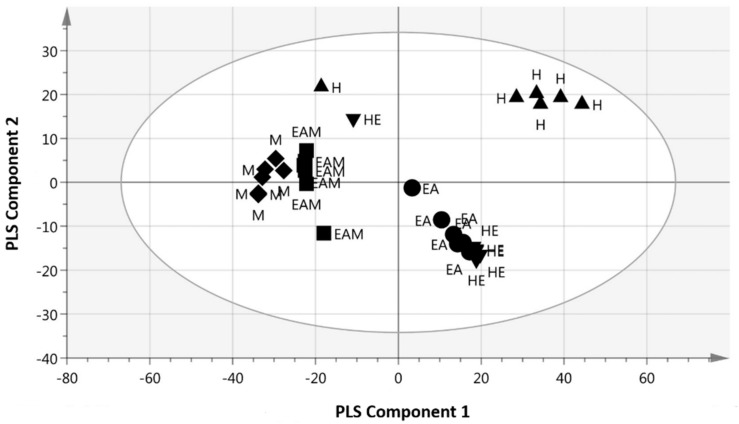
Partial least square (PLS) score scatter plot of different solvent extracts of *C. nutans* leaves.

Figure 2 shows the score scatter plot of the PLS model with good separation of the samples based on the chemical profile and bioactivity (IC_50_ value) of the samples. The high active samples (H, HE, and EA) with the IC_50_ value less than 10 µg/mL were observed at the positive PLS component 1, while the less active samples (EAM and M) were distributed at the negative PLS component 1.

**Figure 3:**
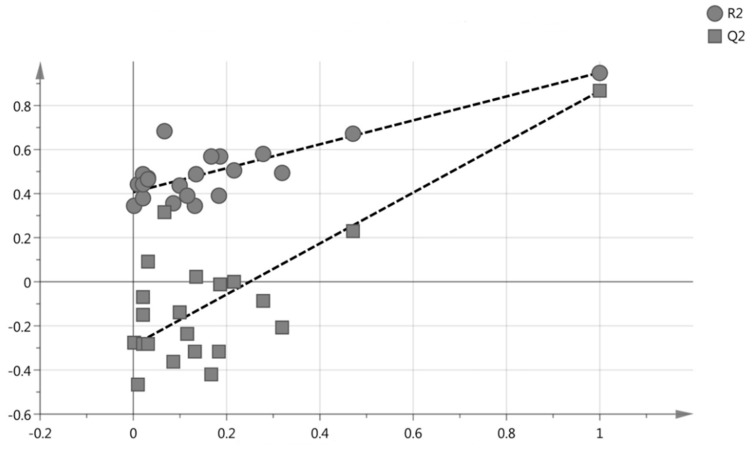
The permutation plot with the R^2^Y = 0.407 and Q^2^Y = −0.288.

Figure 3 displays the permutation test plot, which depicts the validation of the PLS model developed. The plot displays the intercepts for the fraction of the total sum of the squares intercept *Y*-value and the predictive ability of the model intercept *Y*-value. The R^2^Y and Q^2^Y values in this study are 0.407 and −0.288, respectively, which are acceptable since the R^2^ and Q^2^ is equal or less than 0.4 and −0.05, respectively [29]. The supplementary plot, Figure S1 displays the observed versus predicted plot that measures the accuracy of the PLS model with the regression equation that measures its validation with R^2^ value of 0.9497, which is valid only if the R^2^ value is ≥ 0.9.

**Figure 4:**
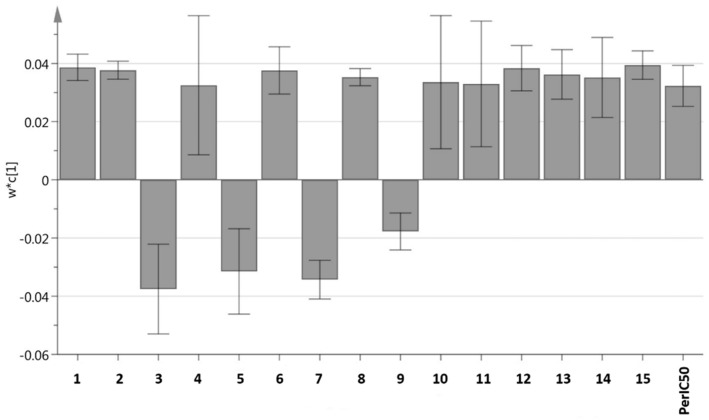
PLS loading column plot of different solvent extracts of *C. nutans* leaves. Assignments: **1**—Palmitic Acid, **2**—Phytol, **3**—Sucrose, **4**—Hexadecanoic acid, **5**—Maltose, **6**—1-Monopalmitin, **7**—d-Glucose, **8**—Stigmast-5-ene, **9**—d-Gluconic acid, **10**—Pentadecanoic acid, **11**—Heptadecanoic acid, **12**—1-Linolenoylglycerol, **13**—Glycerol monostearate, **14**—alpha-Tocospiro B, and **15**—Stigmasterol.

**Figure 5:**
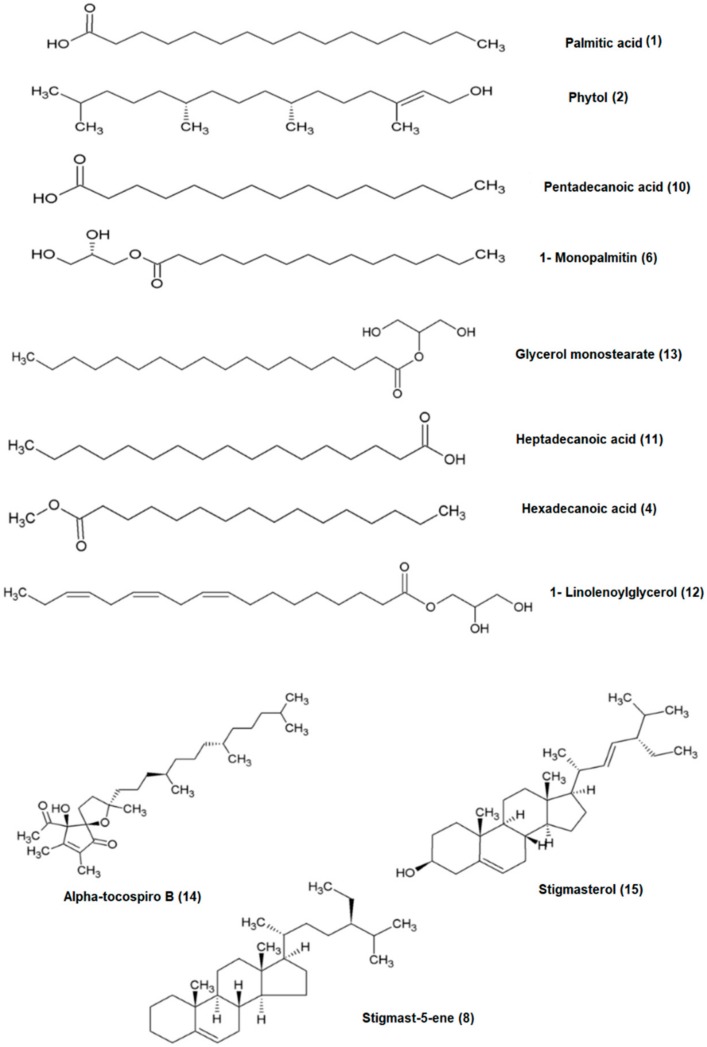
The bioactive compounds that inhibit α-glucosidase enzyme activity.

Figure 4 shows the loading column plot of the different extracts of *C. nutans* leaves. Each peak indicates the identified metabolite from the *C. nutans* leaves extracts and the compounds details have been tabulated in Table 2. The peaks labelled as 3, 5, 7, and 9 represent sucrose, maltose, d-gluconic acid, and d-glucose, respectively, that are located opposite to the per IC_50_ value assumed to induce α-glucosidase activity. These compounds were found abundant in the methanol extract, with its IC_50_ value more than 100 µg/mL. Meanwhile, the rest of the peaks that are in the same position as the per IC_50_ value are effective to inhibit α-glucosidase activity which are primarily plant sterols, fatty acids, organic acids, and other compounds, namely; palmitic acid, phytol, hexadecanoic acid, 1-monopalmitin, stigmast-5-ene, pentadecanoic acid, heptadecanoic acid, 1-linolenoylglycerol, glycerol monostearate, alpha-tocospiro B, and stigmasterol (Figure 5).

### 2.4. Bioactivity Confirmation and Quantification of Some Bioactive Compounds

A further investigation was required to confirm the bioactivity of afore-mentioned identified compounds through testing of the individual compounds on α-glucosidase inhibitory activity. However, not all the compounds could be tested and quantified due to a limitation of the availability of some of these compounds. Therefore, three bioactive compounds were procured and tested. These include palmitic acid, heptadecanoic acid, and stigmasterol. The α-glucosidase inhibitory activity of these compounds was analyzed, and the IC_50_ values were displayed in Table 3. In many cases, the purified compounds were observed to have diminished their bioactivities after separation or purification from the biologically active extract [30]. However, from the results obtained, these compounds showed fairly good α-glucosidase inhibition activity as an individual compound in their purified state.

Quantification of each identified compound through GC-MS was carried out to determine the amount of the identified α-glucosidase inhibitors that is present in the active extract (H) of the *C. nutans* leaves extract. An internal standard, methyl nonadecanoate was used to calculate the recovery during GC-MS analysis. The parameters measured in terms of the linearity, limit of quantification (LOQ), and limit of detection (LOD) are displayed in Table 4. Besides that, the calibration curves established were found to be valid with the coefficient of the regression > 0.99 for all the curves developed for each of the standard references. The recovery of these compounds was calculated to be 93.07%, based on the standard curve developed for the internal standard, MND that represents the three quantified compounds. Overall, the range of LOD values obtained was from 0.31 to 5.00 µg/mg, and the range of LOQ was from 0.63 to 10.00 µg/mg for all the three compounds.

### 2.5. Molecular Docking

Molecular docking was carried out in order to understand the protein-ligand interaction at a molecular level. Generally, protein is comprised of amino acids linked in a sequence to form the complex. These amino acids have different properties depending on their side chains, which can be hydrophobic (aliphatic or aromatic), polar (neutral) or even electrically charged (acidic or basic). This has been the key to the actual properties and behaviors of a protein [31]. All the compounds identified to be actively responsible for the α-glucosidase inhibition were docked to the *Saccharomyces cerevisiae* isomaltase (SCI) crystal structure (PDB ID: 3A4A). The conformations displaying the lowest binding energy for the compounds with the interacting residues are summarized in Table 5 and Table 6. 

**Figure 6:**
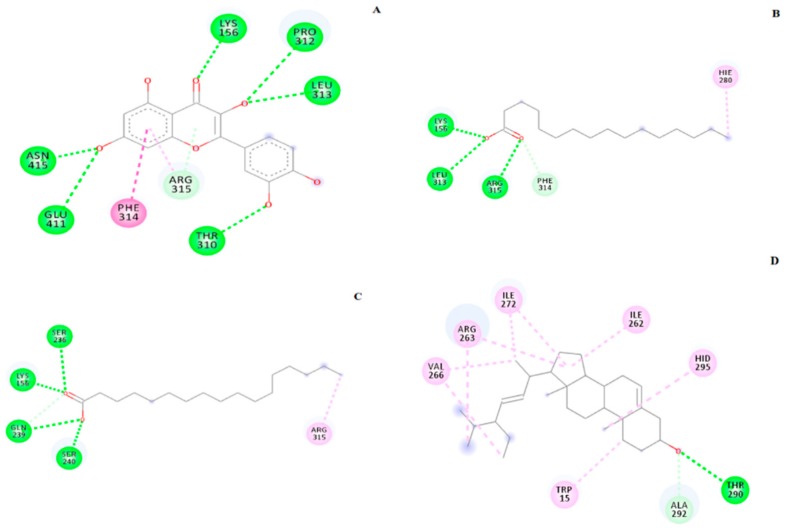
The 2D diagram showing the interaction between the protein residues and the inhibitors. (**A**) Positive control (Quercetin), (**B**) Palmitic acid, (**C**) Heptadecanoic acid and (**D**) Stigmasterol.

**Figure 7:**
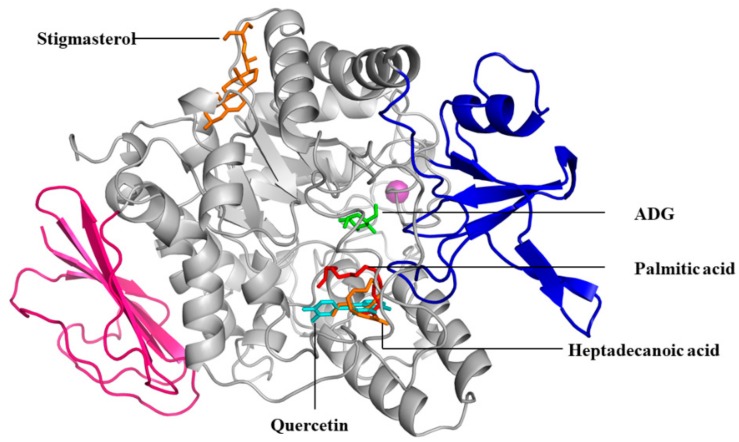
The superimposed 3D diagram showing the binding site of the ADG, Quercetin, Palmitic acid, Heptadecanoic acid and Stigmasterol.

According to AutoDock 1.5.6 simulation result shown in Table 5, the α-glucosidase-quercetin (positive control) inhibitor complex showed −8.15 kcal/mol binding energy containing five hydrogen bonds with the interacting residues viz., ASH69, HIE112, GLN182, ARG213, ASH215, GLH277, HIE351, ASP352, ARG442, and hydrophobic interaction with TYR72. Meanwhile, the binding affinity of palmitic acid in complex with α-glucosidase was −3.75 kcal/mol. A total of three hydrogen bonds were observed in the complex involving LYS156, LEU313, and ARG315 along with hydrophobic interactions involving HIE280 and PHE314. On the other hand, α-glucosidase-heptadecanoic acid complex showed a binding affinity value of −3.80 kcal/mol. Four residues include LYS156, SER236, GLN239, and SER240 were observed to interact with heptadecanoic acid via hydrogen bond, while ARG315 was interacting through π-bond. Despite, interaction with only one residue by hydrogen bond with its hydroxyl group, THR290, stigmasterol has comparably shown a better binding energy of −8.66 kcal/mol than the positive control quercetin. The hydrophobic contacts seemed to be dominant in α-glucosidase-stigmasterol complex due to its cyclic skeleton and alkyl groups that preferably binds to TRP15, ILE262, ARG263, ILE272, VAL266, and HID295. The crystallized ligand, α-d-glucose (ADG) exhibited slightly lower binding energy; i.e., −6.00 kcal/mol, compared to the positive control, quercetin, and stigmasterol. However, the hydrogen bonding between ADG and the protein indicates a stable interaction with a total of nine bonds, involving ASH69, HIE112, GLN182, ARG213, ASH215, GLH277, HIE351, ASP352, and ARG442 with a hydrophobic interaction with TYR72. Unlike the docked ADG, the docking results showed that the binding of the tested compounds are majorly assisted through hydrophobic contacts with α-glucosidase. The results are comparable to those reported by Seong et al., [32] who tested the isoflavones where daidzein, genistein and calycosin showed more hydrophobic contacts with similar residues, such as ILE262, ARG263, ILE272, and VAL266. The 2D diagram showing the interaction between the compounds and the protein residues are shown in Figure 6 while the 3D superimposed diagram of the quercetin, ADG, and three reference compounds (stigmasterol, palmitic and heptadecanoic acid) is displayed in Figure 7.

The binding energy and residues involved in the interaction of other tentative metabolites identified are displayed in Table 6. As for the other α-glucosidase inhibitors identified in this study, stigmast-5-ene and alpha tocospiro B displayed better binding energies; i.e., −9.09 and −7.99 kcal/mol compared to quercetin and ADG. The conformational complex of these compounds is dominantly the hydrophobic contacts with the residues involved; TYR158, LYS156, HIE280, PRO312, PHE314, ARG315, TYR316 and LYS156, TYR158, PHE159, PHE178, HIE280, and PHE314, respectively. In fact, the absence of hydrogen bonding in stigmast-5-ene-α-glucosidase complex may be due to the absence of the hydroxyl group in the inhibitor. The scenario of prominent hydrophobic contacts is also apparent in the other tentative metabolites docked onto α-glucosidase, which is possibly due to the hydrophobicity of the compounds. Overall, the interaction involves hydrogen bonding and other interactions such as C-H, alkyl, and π bond, depending upon the nature of the residues interacting to the atoms as well as the nature of the compounds themselves. All the protein-ligand interactions and the residues involved were comparable to those seen in the docked positive control-α-glucosidase complexes and the validation of molecular docking was done by comparing between the crystal structure of *S. cerevisiae* isomaltase (PDB ID: 3A4A and its docked crystal ligand, ADG [26,33].

## 3. Discussion

The α-glucosidase enzyme can be found in the brush-border of the small intestine where its activity affects the carbohydrate digestion in the human system. During the digestion, starch is broken down to saccharides (oligo- and di-saccharides), which are then hydrolysed to glucose by α-glucosidase before being absorbed into the blood circulatory system. Therefore, the α-glucosidase inhibition is proposed to be an ideal strategy for preventing hyperglycemia. This is because it can prolong the processes of carbohydrate digestion in the intestinal tract, lengthen the duration of glucose absorption, and consequently lead to a lower postprandial blood glucose level [34,35]. Plant metabolites have provided a wide array of pharmacological benefits to human health [36]. In most bioactivity analysis, in vitro techniques are utilized to identify the pharmacological potency of the plant metabolites. Generally, various antidiabetic mechanisms are tested via in vitro procedures, which include α-glucosidase inhibition activity assay as well. The bioactivity of plant metabolites depends on the chemical structure, particularly the number and positions of the active functional groups (e.g., hydroxyl, acetyl) as well as the nature of the substitutions on the aromatic rings [1]. 

In recent times, research using medicinal plants has been an on-going effort due to the vast variety of metabolites distributed in plants that may have various health benefits. Numerous organic solvents and water have been used to extract the potential metabolites from plants. The variation in the solvent polarity resulted in remarkably different bioactivities and phytochemical constituents. Therefore, solvents of different polarity were used in this study to obtain *C. nutans* leaves extracts to evaluate the different metabolites that may have different polarity thus exhibiting different potential in the α-glucosidase inhibition assay analysis. The results exhibited an increasing activity with decreased polarity significantly thus indicating the presence of compounds of less polar nature such as the terpenoids, sterols and fatty acids group members that are responsible for the inhibition activity [37]. The outcome was influenced by the variability of the metabolites extracted using solvents with different polarity. 

Some of the identified compounds have already been reported to possess α-glucosidase inhibitory activity. Liu et al. [38] carried out an investigation on the α-glucosidase inhibitory activity of some fatty acids including palmitic acid. However, the IC_50_ value of palmitic acid was reported > 400 µg/mL which was much higher than the value obtained in our study. Apart from palmitic acid, other fatty acids that are found to be active inhibitors include pentadecanoic acid, heptadecanoic acid, and hexadecanoic acid (methyl ester). Marella et al. [39] reported these fatty acids in the *C. vitalba* formulation, which showed strong antidiabetic activity. Another compound identified to inhibit the α-glucosidase enzyme activity is phytol. Phytol is an acyclic diterpene alcohol, commonly referred to as isoprenoids, which is generally produced from the degradation of chlorophyll. The same compound has already been reported to exhibit strong antinociceptive and antioxidant activities [40]. In a report by Tundis et al. [41], it was shown that phytol is the most prevailing component contributing to the strong α-glucosidase inhibiting activity of *C. annuum* of *cerasiferum* variety fruits’ hexane fraction. 

Plant sterols and its chemical and structural derivatives are secondary metabolites that can be found ubiquitously in plant parts. Some of the group compounds that were identified in this plant extract include 1-linolenoylglycerol, glycerol monostearate, and stigmasterol. These compounds are found to be active against α-glucosidase enzyme in this study. Stigmasterol has been reported to potentially exhibit hypoglycemic activity by effectively reducing serum glucose level as well as the activity of hepatic glucose-6-phophatase (G-6-P) with a concomitant increase in insulin concentration in in-vivo study [42,43]. Besides, stigmasterol isolated from *Bacopa monnieri* Linn. aerial parts was reported to have insulin-like activity where its antihyperglycemic effect was suggested to increase the peripheral glucose consumption as well as protection against oxidative damage in alloxanised diabetes, which also indicated its antioxidant potential [44]. However, Zareen et al. [45] concluded that the stigmasterol isolated from *Cichorium intybus* showed no inhibitory activity against α-glucosidase. This may be due to the antagonism effect caused by the trace impurities in the isolated compound, or due to the difference in assay conditions. 

Another compound that belongs to the family of monoacylglycerols is 1-monopalmitin (6), which is a glyceride consisting of one fatty acid chain covalently bonded to a glycerol molecule through an ester linkage. This study shows that this compound is capable of inhibiting α-glucosidase. The compound consists of two hydroxyl groups that might contribute to the bioactivity (HMDB) [46]. To our knowledge, there is no scientific report that has ever been published on the α-glucosidase inhibitory activity of this compound, despite its structural compatibility. 

The other compounds that have been found to inhibit α-glucosidase enzyme activity in our study include stigmast-5-ene, 1-linolenoylglycerol, glycerol monostearate, and alpha-tocospiro B. However, from the literature, no report has emerged for the α-glucosidase inhibiting activity of these compounds. Meanwhile, the rest of the compounds identified are the ones assumed to be effectively contributing to induce α-glucosidase activity via a special mechanism. These compounds include sucrose, maltose, d-glucose, and d-gluconic acid, which was found inactive in our study. 

Despite being claimed by previous studies for its low activity in α-glucosidase inhibition assay, palmitic acid and stigmasterol in this study have exhibited a good activity. The potential of these two compounds shall be further investigated for their use in the development as effective α-glucosidase inhibitors. Hence, the plant leaves extract, due to the presence of aforementioned α-glucosidase inhibitors, in a significant quantity has a great potential to be used as an efficacious anti-hyperglycemia remedy. 

Generally, all known α-glucosidase inhibitors possess multiple hydroxyl groups that become the reason for their high activity [32,33]. However, all the compounds identified in our study are non-polar compounds comprising of sterols, fatty acids, monoacylglycerols, and terpene alcohol. These compounds are known to possess a minimal amount of the hydroxyl group one or two [47]. Despite this reason, the tested compounds are regarded to have α-glucosidase inhibiting activity based on the findings above. Moreover, the interactions between the protein and the inhibitors have suggested the possible conformational complexes that may have caused the positive enzyme inhibitory activity of the identified compounds.

From the results, it can be deduced that apart from the strong hydrogen bond between the hydroxyl group of the compounds with the residues possessing polar side chains (GLN and SER) and electrically charged side chains (ARG, ASP and HIE- protonated HIS), the hydrophobic and π-interactions between the compounds cyclic part of the structure and hydrophobic side chains (TYR, VAL, ILE and PHE) of the enzyme could explain the much higher activities of these non-polar compounds, which is agreeable to the finding reported by Nussinov [48] and Du et al. [49]. This can be observed in quercetin as well as other compounds. The residues interacted with ADG and its binding energy is contributing to its actual catalytic reaction [32,50]. As shown in Table 5, ADG interacted with ASH215, a conserved residue that acts as the catalytic nucleophile. It also created a hydrogen bond with GLH277 (protonated GLU) residue that acted as the general acid-base catalysis. In addition, ADG also interacted with ASP352, which is responsible for stabilizing the substrate during the catalysis in order to strengthen the acid-base hydrolysis reaction [32,50]. 

Besides that, the 3D diagram (Figure 7) also showed that the protein may possess more than one ligand-binding site, which can offer allosteric control for catalytic activity. This may cause the alteration to the active site and thus result in the inhibition of the enzyme activity. Allosteric inhibitors have the ability to fine-tune protein functional activity [49,51]. Generally, allosteric binding ability reflects the conjugated behavior where conformational perturbations elicited at one region in a macromolecule affect its active site and thus alters the enzyme activity. Occasionally, any unbound conformation such as allosteric interactions might be unstable; however, the binding residues may eventually stabilize it. This may result in the equilibrium shifting toward this conformer or the allosteric ligand that may display an altered functional site shape [48,52]. 

Moreover, the superimposed 3D diagram (Figure 7) also suggested that the tested compounds may exhibit non-competitive inhibition mode compared to the control ligand, ADG, a known competitive inhibitor [32]. Stigmasterol was observed to be bound away from the active site. Its binding is dominantly assisted through hydrophobic contacts that contribute to its binding energy thus reflecting its inhibition activity. This phenomenon may possibly cause conformational changes in the active site of the enzyme, which may slow down the protein activity [33,48,52]. The stigmasterol’s predicted binding area could be a promising region for inhibiting this protein activity overall through a non-competitive mode can be compared with compounds of a similar nature. In addition, due to a lack of hydroxyl groups in the tested compounds, the hydrophobic and π-interactions apparently play a greater role for these compounds’ enzyme inhibitory activity. The binding site of the control ligand on α-glucosidase was close to the active site pocket, while the others were outside of the pocket indicating the presence of allosteric binding sites that may possibly alter the enzyme activity. This can be observed in palmitic acid, heptadecanoic acid, and quercetin binding regions (Figure 7). This arrangement might induce cleft closure to prevent the entrance of the substrate, which subsequently acts as the non-competitive inhibiting nature of the metabolites [53]. Generally, these types of inhibitors have several advantages over the competitive ones since their effect lasts for longer periods of time [54]. It can be highlighted here that, apart from the quantified inhibitors, stigmast-5-ene and alpha-tocospiro B (binding energies of −7.99 and −9.09 kcal/mol, respectively) showed promising potential as allosteric in a non-competitive inhibition manner with high hydrophobicity and good binding energy for the hydrophobic contacts made. 

Furthermore, the docking result is also agreeable to what has been reported by Zheng et al. [55] in their very recent study. The tetracyclic xanthone derivatives that were docked into *S. cerevisiae* α-glucosidase enzyme also resulted in similar hydrophobic and π-interactions between the ligand and protein observed. Apart from that, another study on docking conducted by Ali et al. [33] on two plastoquinones, namely sargachromenol and sargaquinoic acid isolated from the active n-hexane fraction of *S. serratifolium*, (the IC_50_ value of 42.41 and 96.17 µM respectively), exhibited dominantly hydrophobic interactions suggesting that these compounds are allosteric inhibitors against α-glucosidase. Conclusively, molecular docking in this study has provided supportive data for α-glucosidase inhibition allowing the prediction of the interaction between α-glucosidase and tested inhibitors. This can help understanding and virtually visualize the actual scenario of the bioassay performed at molecular level. Further investigation on other tentative metabolites for their bioassay activity will be an added advantage to confirm their actual mechanism.

## 4. Materials and Methodology

### 4.1. Materials

Organic solvents of analytical grade were purchased from Merck (Frankfurter Strasse, Darmstadt, Germany). The α-glucosidase (yeast maltase; *Saccharomyces cerevisiae*) was purchased from Megazyme (Bray Co., Wicklow, Ireland). The substrate ρ-Nitrophenyl-ρ-d-glucopyranosidase (PNPG), *N*-methyl-*N*(trimethylsilyl) trifluoroacetamidepurum (97.0%) (GC grade), all reference compounds (quercetin, palmitic acid, heptadecanoic acid and stigmasterol) and internal standard compounds (methyl nonadecanoate) were obtained from Sigma-Aldrich (St. Louis, MO, USA).

### 4.2. Sample Collection

Matured leaves of *C. nutans* were harvested from Ees Biotech Sdn. Bhd. located at Bukit Mertajam, Penang, Malaysia. The plant sample was submitted for authentication to the Herbarium at International Islamic University Malaysia, Kuantan Campus, Malaysia. The specimen’s voucher number is PIIUM 0238-1. The leaves were washed and dried under shade at room temperature (25 ± 0.5 °C) for a week. The dried leaves were then coursed to powder by using an universal cutting mill (Fritsch, Idar-Oberstein, Rhineland-Palatinate, Germany) and stored at −80 °C prior to extraction [56]. 

### 4.3. Preparation of Extract

The dried *C. nutans* leaves were macerated in 80 % methanol using the sample to solvent ratio of 1:3 (*w*/*v*) for 3 days where the solvent was changed each consecutive day. The crude extract was carefully filtered using Whatman filter paper No.1 followed by solvent evaporation using a rotary evaporator (Büchi, Flawil, Switzerland) under reduced pressure at 40 °C. Further maceration was subjected to the dried crude extracts using different solvents with different polarities, ranging from hexane, hexane: ethyl acetate (1:1, *v*/*v*), ethyl acetate, ethyl acetate: methanol (1:1, *v*/*v*), and finally methanol with the sample to solvent ratio of 1:3 (*w*/*v*). Again, all the extracts were then filtered and dried using rotary evaporator followed by freeze drying process to remove the remaining moisture. The extracts were prepared in six replicates and stored at −80°C prior to further analysis. Each extract was tested for α-glucosidase inhibitory activity and analyzed using GC-MS [56,57]. 

### 4.4. α-Glucosidase Inhibition Assay

The α-glucosidase inhibitory activity assay was performed based on the protocol mentioned by Javadi et al. [1] which mimics the condition of intestinal fluid. Quercetin (1 mg/mL) was used as a positive control, a known α-glucosidase enzyme inhibitor, and ρ-Nitrophenyl-ρ-d-glucopyranosidase (PNPG, 0.3 mg/mL) was used as substrate. Accurately, 10 µL of the samples, quercetin, and negative control (solvent) were transferred into 96-well plate. The reaction mixture of samples and control contain 100 µL of 30 mM phosphate buffer and 15 µL of α-glucosidase (0.02 U/µL) type one from *Saccharomyces cerevisiae*. Meanwhile the blank consists of 115 µL of the buffer without the enzyme. Both sample and blank mixture were then treated with 75 μL of PNPG after 5 min of incubation at room temperature. The reaction was stopped using glycine (pH 10) after another 15 min of incubation. The amount of ρ-nitrophenol released from PNPG was measured using microplate reader (Tecan Nanoquant Infinite M200, Mannedorf, Zurich, Switzerland) at 405 nm. The half-maximal inhibition concentration (IC_50_) value was determined using linear regression analysis based on the absorbance of the stock solution dilutions for both samples and controls. The determination of IC_50_ value is an informative measure of a reactant’s efficacy and potency whereby it indicates the amount of the reactant or inhibitor required to reduce the response by half [37]. All determination was done in triplicates. The inhibitory activity (%) of each extract was calculated using the following formula:

Inhibitory activity (%) = [(A_control_ − A_sample_)/A_control_] × 100% (1)

where, A_control_ is the absorbance of the negative control, and A_sample_ is the absorbance of sample or positive control.

### 4.5. Derivatization Procedure

The samples were derivatized prior to injection into GC-MS as described by Javadi et al. [1]. The sample extract of 25 mg was weighed in a 2 mL centrifuge tube, dissolved in 50 μL of pyridine and sonicated (Elma, S 30H Ultrasonic, South Orange, NJ, USA) for 10 min at 30 °C. As much as 100 μL of methoxyamine HCl (20 mg/mL in pyridine) were added to the sample solution and vortexed. The mixture was then incubated for 2 h at 60 °C in an incubator shaker (Innova 4000-M1192, Weender Landstr, Goettingen, Germany). Then, the tube was incubated for another 30 min at 60 °C after the addition of 300 μL of *N*-Methyl-*N*-(trimethylsilyl) trifluoroacetamide (MSTFA). Finally, the sample solution was filtered using a syringe filter and covered with aluminum foil and left to stand overnight at room temperature (25 ± 0.5 °C) prior to injection into the GC-MS. 

### 4.6. GC-MS Analysis

GC-MS analysis was performed following the method described by Javadi et al. [1] with slight modification. One micro liter of derivatized sample was injected in the split mode with the ratio of 10:1 into the GC-MS system, which consisted of an Agilent 6890 GC-MS (Agilent Technologies, Santa Clara, CA, USA) and a HP 5973 mass selective detector (Agilent Technologies). The GC column used for the analysis was a DB-5MS 5% phenyl methyl siloxane column with an inner diameter (ID) of 250 μm and a film thickness of 0.25 μm (Agilent Technologies, Santa Clara, CA, USA). The initial oven temperature was set to 190 °C for 30 min, and then increased to a target temperature of 300 °C in 10 min at a rate of 5 °C/min and again raised to 320 °C in 10 min at a rate of 10 °C/min. Helium was used as the carrier gas with flow rate of 1 mL/min. The injector and ion source temperatures were set to 250 and 280 °C, respectively. Mass spectra were acquired using a full scan and a monitoring mode with a mass scan range of 50 to 550 *m*/*z*. The spectra for each of the chromatogram peaks were compared with those in the NIST14 database library. The chromatogram and mass spectra were processed using an Agilent ChemStation, Automated Mass Spectral Deconvolution and Identification System (AMDIS) and Agilent’s Deconvoluted Reporting Software (DRS). 

Data pre-processing was carried after GC-MS analysis where all raw chromatograms were converted into cdf format using ACD/Spec Manager v.12.00 (Advanced Chemistry Development, Inc., ACD/Labs Ontario, Toronto, ON, Canada). A further pre-processing procedure extracted all the related information from the raw data and converted it to a data matrix, which includes systematic noise filtering, automatic peak detection, baseline correction, data binning, deconvolution, and chromatographic alignment were done using MZmine software (version 2.3, Okinawa Institute of Science and Technology Graduate University, Kunigami-gun, Okinawa, Japan) [58]. Finally, the data were summarized into an Excel sheet form prior to multivariate data analysis.

### 4.7. Quantification of Active Phytoconstituents

Quantification of the active inhibitory compounds was carried out using the GC-MS according to the methods described by Salimon et al. [59] and Shailajan et al. [60] with some modifications. The three reference compounds that were available readily in-house were palmitic acid (S1), heptadecanoic acid (S2) and stigmasterol (S3). Quantification of each identified compound was carried out to determine the amount of the identified α-glucosidase inhibitors present in the active extract of the *C. nutans* leaves extract. The parameters measured in terms of the linearity, limit of quantification (LOQ) and limit of detection (LOD) are displayed in Table 4. The LOD and LOQ were determined by ratio of noise to signal, 1:3 and 1:10 respectively. 

#### 4.7.1. Development of Standard Curve of the Reference

Firstly, the standard curve was developed for the reference compounds (S1, S2 and S3). The derivatized reference solutions were prepared by dissolving 1 mg in 50 µL pyridine and 100 µL methoxyamine hydrochloride that was added to the mixture before it was allowed to incubate at 60 °C for 2 h prior to the addition of 300 µL of MSTFA to complete the derivatization procedure. The stock solution of the derivatized reference was further diluted using pyridine with a dilution factor of 2 (1 mg/450 µL, 0.5 mg/450 µL, 0.25 mg/450 µL and 0.125 mg/450 µL derivatized solution) before the injection of 1 µL of the solution into GC-MS. The standard curve was prepared based on the peak area of the reference from the resulting chromatogram, whereby a linear regression equation was obtained (y = mx + c), with its linearity (R^2^) ≥ 0.90. 

#### 4.7.2. Determination of LOD and LOQ Concentration 

Limit of detection (LOD) and limit of quantification (LOQ) were also determined for the reference compounds and the internal standard right after the series of dilution analyses. Each reference compound was prepared by dissolving 1 mg of the reference in 8 µL pyridine and 16 µL of methoxyamine hydrochloride added and followed by 48 µL of MSTFA after the incubation period. All three derivatized references were mixed and further diluted with a dilution factor of 2 (0.5 mg/72 µL, 0.25 mg/72 µL, 0.125 mg/72 µL derivatized solution, etc.) prior to injection into GC-MS. LOD and LOQ were determined by ratio of noise to signal, 1:3 and 1:10, respectively.

#### 4.7.3. Determination of Recovery of the Internal Standard (Methyl Nonadecanoate)

##### Development of Standard Curve of the Internal Standard

The derivatized internal standard solution was prepared following the routine derivatization method mentioned in Section 2.5. The stock solution of the derivatized internal standard was further diluted using pyridine with a dilution factor of 2 (12.5 mg/450 µL, 0.5 mg/450 µL, 0.25 mg/450 µL, and 0.125 mg/450 µL derivatized solution) and each was injected into the GC-MS. The standard curve was then prepared based on the peak area of the internal standard from the chromatogram with a regression line possessing linearity of more than 0.90. 

##### Calculation of the Recovery of the Internal Standards

In order to calculate the recovery of the internal standard (IS) in the sample representing the three quantified compounds overall, the plant sample was spiked with the internal standard and derivatized before GC-MS analysis. About 24 mg of the plant extract (hexane) was weighed with 1 mg of the internal standard (W_1_) dissolved in 50 µL pyridine and further treated following the procedure as mentioned above. About 1 µL of the derivatized reference solution was injected into GC-MS and the recovered amount of the internal standard (W_2_) was calculated by substituting its peak area into the regression equation obtained from its standard curve. The compound recovery was reported as percentage of recovery following the equation below:

R (%) = (W_2_/W_1_) × 100% (2)

#### 4.7.4. Determination of Targeted Metabolites Concentration in the Sample

The concentrations of the targeted metabolites in the plant extract were determined by preparing the derivatized plant extract as per above with 25 mg of the extract and 1 µL of the prepared extract was injected into GC-MS. The peak area of each of the targeted metabolites was obtained from the chromatogram and the concentration (x) was calculated using the regression equation developed from the reference standard curve by substituting the peak area [60].

### 4.8. Molecular Docking

A 1.6 Å resolving crystallographic structure of yeast α-glucosidase; *Saccharomyces cerevisiae* isomaltase (SCI) obtained from Protein Data Bank (PDB) (http://www.rcsb.org/pdb/explore/explore.do?structureId=3a4a; PDB code: 3A4A) was selected as the receptor in this molecular docking. The receptor was selected based on the sequence similarity of its amino acid residues between the target protein and the template (3a4a) was reported to be 71.92%. The 3D structure of positive control (quercetin) and active compounds identified in GC-MS metabolomics multivariate analysis was obtained from Pubchem (National Center for Biotechnology Information (NCBI), Bethesda, MD, USA) and Chemspider (Royal Society of Chemistry, Milton Road, Cambridge, UK). All the water molecules were removed from the protein structure using the AutoDockTools (The Scripps Research Institute, La Jolla, CA, USA) of the version 1.5.6 [61]. Prior to the molecular dockings, the homology model of the enzyme was developed according to the assay condition with pH of 6.5 using PDB2PQR Server, version 2.0.0 (National Biomedical Computation Resource, San Diego, CA, USA) [62] in order, to mimic the actual condition of the mechanism medium. The docking grid box parameter was set to cover an area of 126 Å × 126 Å × 126 Å and centered to the protein at coordinate x, y, and z of 21.272, −0.751 and 18.633, respectively. For the ligands, Gasteiger charges were added, while the rotatable bonds in the ligand were assigned with AutoDockTools and all torsions were allowed, to rotate. The docking jobs were performed with AutoDock (version 1.5.6, The Scripps Research Institute, La Jolla, CA, USA) using the Lamarkian genetic algorithm [63]. A population size of 150 and energy evaluations of 2,500,000 (medium) were set to generate 50 ligand docked conformations [26,32,53,64]. The 2D diagram showing the interactions between the protein residues and the compounds were generated using Biovia Discovery Studio (Biovia, San Diego, CA, USA) while the 3D superimposed diagram of the complex was rendered using PyMOL™ 1.7.4.5 (Schrödinger, LLC, New York, NY, USA).

### 4.9. Data Processing and Statistical Analysis

The data were represented as mean ± standard deviation (SD), with six replicates using Minitab 17 (Minitab Inc., State College, PA, USA). The *p* value was obtained from the ANOVA analysis using the Tukey’s test with *p* < 0.05 was considered significant. One-way ANOVA with a Tukey comparison test was used to evaluate the major differences between the samples with a confidence interval of 95%. As for the multivariate data analysis, the pre-processed data were fed to the SIMCA P^+^14.0 software (version 14.0, Umetrics, Umeå, Västerbotten, Sweden) using Partial Least Square (PLS) applying the UV scaling method. The model was fitted and validated through the permutation test. Finally, the score scatter and loading plots were produced. 

## 5. Conclusions

This study investigated the potential antidiabetic activity of different *C. nutans* extracts that focused on the inhibitory effects of α-glucosidase enzyme carried out based on GC-MS. The hexane extract exhibited remarkable inhibitory activity. Some of the potential inhibitor compounds in this extract were identified and docked to show their molecular interactions that supported the overall results. The molecular interactions of the inhibitors identified with the protein were predominantly hydrogen bonding involving residues namely LYS156, THR310, PRO312, LEU313, GLU411, and ASN415 as well as PHE314 and ARG315 residues with hydrophobic interaction. Conclusively, the docking results have shown the inhibiting activity of the inhibitors as allosteric binders thus acting as non-competitive inhibitors. The overall results of this study provide scientific evidences that exhibit the potential of *C. nutans* in α-glucosidase enzyme inhibition, which can be a contribution to the development of medicinal preparations, nutraceuticals, or functional foods for diabetes and related symptoms as well as for further exploration of the plant’s novel therapeutic or preventive agents for the treatment of diabetes.

## Figures and Tables

**Table 1:** The half maximal inhibitory concentration (IC_50_) of α-glucosidase inhibitory activity of *C. nutans* leaves extracts.

Samples	IC_50_ (μg/mL)
Hexane (H)	3.05 ± 0.05 ^d^
Hexane: Ethyl acetate (HE)	5.54 ± 0.09 ^d^
Ethyl acetate (EA)	8.42 ± 0.17 ^c^
Ethyl acetate: Methanol (EAM)	37.45 ± 0.90 ^b^
Methanol (M)	133.57 ± 0.30 ^a^
Quercetin	5.77 ± 1.01 (19.09 μM)

Data expressed as mean ± SD (*n* = 6). SD = Standard Deviation. Means that do not share a letter are significantly different with *p* value < 0.05.

**Table 2:** Compounds identified in the *C. nutans* leaves extracts through GC-MS analysis.

No.	RT (min)	% of Area	Probability	M^+^	Molecular Formula	Tentative Metabolites
**1**	17.24	10.50	99	256.24	C_16_H_32_O_2_	Palmitic acid
**2**	26.64	1.42	95	296.31	C_20_H_40_O	Phytol
**3**	45.47	36.18	93	342.12	C_12_H_22_O_11_	Sucrose
**4**	11.63	0.91	99	270.26	C_17_H_34_O_2_	Hexadecanoic acid
**5**	49.48	0.28	93	342.12	C_12_H_22_O_11_	Maltose
**6**	44.43	1.03	95	330.51	C_19_H_38_O_4_	1-Monopalmitin
**7**	10.31	19.04	91	180.06	C_6_H_12_O_6_	d-Glucose
**8**	56.83	4.50	99	398.39	C_29_H_50_	Stigmast-5-ene
**9**	14.30	3.42	93	196.16	C_6_H_12_O_7_	d-Gluconic acid
**10**	12.34	0.24	98	242.22	C_15_H_30_O_2_	Pentadecanoic acid
**11**	23.95	0.38	93	270.26	C_17_H_34_O_2_	Heptadecanoic acid
**12**	47.59	0.46	99	352.52	C_17_H_36_O	1-Linolenoylglycerol
**13**	47.99	0.40	91	358.31	C_21_H_42_O_4_	Glycerol monostearate
**14**	49.24	0.07	96	462.37	C_29_H_50_O_4_	Alpha-Tocospiro B
**15**	55.59	2.70	99	412.37	C_29_H_48_O	Stigmasterol

RT = Retention Time C = Carbon, H = Hydrogen, O = Oxygen, N = Nitrogen.

**Table 3:** The half maximal inhibitory concentration (IC_50_) of α-glucosidase inhibitory activity of quantified reference compounds.

Samples	IC_50_ Value	
	µg/mL	µM
Palmitic acid	24.09 ± 0.44 ^c^	93.95
Heptadecanoic acid	26.10 ± 0.77 ^b^	96.51
Stigmasterol	65.31 ± 0.37 ^a^	158.25
Quercetin	5.77 ± 1.01	19.09

Data expressed as mean ± SD (*n* = 6). SD = Standard Deviation. Means that do not share a letter are significantly different with *p* value < 0.05.

**Table 4:** Calibration curves, detection limits and quantification limits measured by GC-MS.

Analytes	R^2 1^	Concentration (µg/mg Sample) ^2^	LOD (µg/mg Sample)	LOQ (µg/mg Sample)
Palmitic acid	0.99	433.25 ± 22.96 ^a^	2.50	5.00
Heptadecanoic acid	0.99	20.27 ± 1.20 ^c^	5.00	10.00
Stigmasterol	0.99	386.48 ± 21.03 ^b^	0.31	0.63

LOD = Limit of Detection; LOQ = Limit of Quantification. ^1^ R^2^ is the correlation coefficient of the equation with *n* = 3. ^2^ Data is expressed as mean ± SD (*n* = 6), calculated considering % recovery of methyl nonadecanoate (≥98.0%). Means that do not share a letter are significantly different with *p* value < 0.05.

**Table 5:** Molecular interaction results of α-glucosidase enzyme protein with the known inhibitor (Quercetin) and the active compounds quantified using GC-MS.

Compound	Binding Energy (kcal/mol)	H-bond Interacting Residues	Other Interacting Residues
Control ligand	−6.00	ASH69, HIE112, GLN182, ARG213, ASH215, GLH277, HIE351, ASP352, ARG442	TYR72
Quercetin	−8.15	LYS156, THR310, PRO312, LEU313, GLU411, ASN415	PHE314, ARG315
Palmitic acid	−3.75	LYS156, LEU313, ARG315	HIE280, PHE314
Heptadecanoic acid	−3.80	LYS156, SER236, GLN239, SER240	ARG315
Stigmasterol	−8.66	THR290	TRP15, ILE262, ARG263, ILE272, VAL266, HID295

**Table 6:** Molecular interaction results of other tentative metabolites docked onto α-glucosidase enzyme.

Compound	Binding Energy (kcal/mol)	H-bond Interacting Residues	Other Interacting Residues
1-linolenoylglycerol	−4.76	LYS156, SER241	TYR158, SER240, PHE314
1-monoplamitin	−4.01	TYR158, SER240, ASP242	PHE314, ILE419
Alpha-tocospiro B	−7.99	GLN279, GLU411	LYS156, TYR158, PHE159, PHE178, HIE280, PHE314
Glycerol monostearate	−3.95	SER241	LYS156, TYR158, SER240, ASP242, PHE314, ARG315, TYR316
Hexadecenoic acid (methyl ester)	−4.00	ALA292, ASN259, HID295	VAL266
Pentadecanoic acid	−4.42	HID295	VAL266
Phytol	−5.51	GLN239, SER240	LYS156, TYR158, VAL232, LEU313, PHE314
Stigmast-5-ene	−9.09	-	TYR158, LYS156, HIE280, PRO312, PHE314, ARG315, TYR316

## Supplementary Materials

The following is available online. Figure S1: Prediction versus observation of IC_50_ values from all sample. The R^2^ of the regression line indicates the goodness of fit between experimental observations and predicted model.
