# Supplementary material for: Characterization of α-Glucosidase Inhibitors from Clinacanthus nutans Lindau Leaves by Gas Chromatography-Mass Spectrometry-Based Metabolomics and Molecular Docking Simulation
Source: Molecules. 2018 Sep 19;23(9):2402. doi: 10.3390/molecules23092402 (PMC6225325; doi:10.3390/molecules23092402)
Supplement: Supplementary file 1 [file molecules-23-02402-s001.pdf]

Clinacanthus nutans Lindau Leaves by Gas Chromatography-Mass Spectrometry-Based Metabolomics and Molecular Docking Simulation

Suganya Murugesu [1], Zalikha Ibrahim [1], Qamar-Uddin Ahmed [1], Nik-Idris Nik Yusoff [1], Bisha-Fathamah Uzir [1], Vikneswari Perumal [2], Faridah Abas [3], Khozirah Saari [3], Hesham El-Seedi [4,5] and Alfi Khatib [1,3,*]

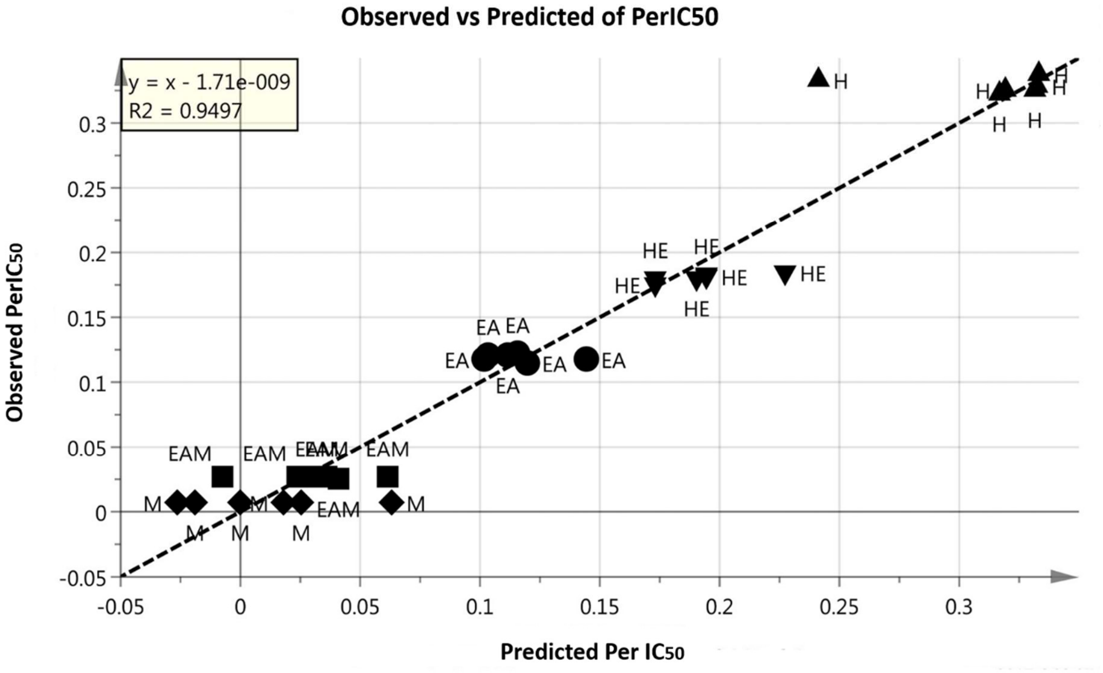

Figure S1. Prediction versus observation of IC_{50} values from all sample.
